# An Unusual Presentation From a Sporadic Partially Acid-Fast Aerobic Actinomycete Resistant to Common Antibiotics

**DOI:** 10.1177/2324709619899598

**Published:** 2020-01-23

**Authors:** Sreedhar Adapa, Srikanth Naramala, Vijay Gayam, Rajan Kapoor, Mina Raju, Pallav Patel, Venu Madhav Konala

**Affiliations:** 1 The Nephrology Group, Fresno, CA, USA; 2 Adventist Medical Center, Hanford, CA, USA; 3 Interfaith Medical Center, Brooklyn, NY, USA; 4 Augusta University Medical Center, Augusta, GA, USA; 5 Kaweah Delta Medical Center, Visalia, CA, USA; 6 Ashland Bellefonte Cancer Center, Ashland, KY, USA

**Keywords:** *Nocardia*, *Nocardia farcinica*, immunocompromised, peritonitis

## Abstract

Nocardia causes rare opportunistic infections, that can be challenging to diagnose because of atypical features on conventional microbiological identification techniques. Immunosuppressed patients are more susceptible to infections from *Nocardia* and are associated with multi-organ involvement. We report a case of a 63-year-old male who developed peritonitis from *Nocardia farcinica* that rarely causes infections in humans. The nonspecific symptoms, negative blood cultures, and slow growth can make diagnosis difficult. Despite aggressive therapy, the virulence and inherent resistance to the antibiotics can result in high mortality from *Nocardia farcinica* infections.

## Introduction

*Nocardia* species are opportunistic organisms, which can rarely cause infections in humans.^1^ Peritonitis is encountered in the patients undergoing peritoneal dialysis and often can be severe. Improved microbiological techniques are being frequently used to identify the uncommon organisms causing peritonitis.^2^ We present a case of peritonitis from *N farcinica*, which was reported only once in literature before.^3^

## Case Report

A 63-year-old male on peritoneal dialysis presented to the home dialysis clinic with a chief complaint of cloudy effluent. The patient was started on empiric intraperitoneal vancomycin and gentamicin for suspected peritonitis as the patient was allergic to penicillin. The patient developed severe itching, rash, and dry heaves after 10 minutes of intraperitoneal antibiotics dwell and was drained. The patient was sent to the emergency room for further management. The patient also had bilateral lower extremity cellulitis for 2 weeks, which was not getting better with oral cefalexin as an outpatient. He also admitted that he was not able to ambulate for a week and was having excruciating pain in the lower extremities on ambulating only short distances. The patient was admitted to the hospital for management of peritonitis and cellulitis. He was started on intravenous daptomycin and aztreonam.

The past medical history was significant for atrial fibrillation, type 2 diabetes, coronary artery disease, coronary artery bypass grafting, end-stage renal disease on peritoneal dialysis, mechanical mitral, and aortic valve repair on anticoagulation, implantable cardioverter-defibrillator. Outpatient medications include alprazolam 2 mg twice a day, bupropion 150 mg twice a day, calcitriol 0.25 µg daily, carvedilol 25 mg twice a day, furosemide 80 mg daily, losartan 100 mg daily, simvastatin 80 mg daily, and warfarin 3 mg daily. The patient was an ex-smoker and denied any intake of alcohol or drugs. Physical examination showed implantable cardioverter-defibrillator placement, has a mechanical click at the left lower sternal border, soft systolic ejection murmur, peritoneal dialysis catheter in place with no exit site infection, and bilateral lower extremity ulcerations, which had an eschar with surrounding erythema and were exquisitely tender on palpation.

The peritoneal fluid analysis sent from the dialysis clinic revealed 206 nucleated cells, 5% segmented neutrophils, 90% lymphocytes, and 5% eosinophils. The antibiotics were continued for 2 days in the hospital and were stopped as there was no growth in the peritoneal dialysis fluid, and repeated cell counts remained insignificant. The infectious disease specialist actively followed the patient since the day of admission to the hospital. During the hospitalization, the patient was evaluated by the vascular surgeon for nonhealing lower extremity ulcers. The patient was started on sodium thiosulfate for suspected calciphylaxis. The patient underwent lower extremity angiogram with right anterior tibial artery angioplasty for excruciating right lower extremity pain, which was cold and appeared necrotic. The patient developed retroperitoneal hematoma post angioplasty, secondary to supratherapeutic international normalized ratio, and the patient was managed conservatively by stopping warfarin for 3 days and administering fresh frozen plasma. The pain became unbearable for the patient despite angioplasty, and the patient underwent right above knee amputation. The patient subsequently developed left lower extremity pain and blisters during the hospital stay, and the patient underwent angiogram with angioplasty and recanalization of the totally occluded left anterior tibial artery. The vascular interventions and retroperitoneal hematoma prolonged the hospital stay. The patient continued to receive renal replacement therapy through peritoneal dialysis.

One month into the hospital admission, the peritoneal fluid was analyzed for suspicion of peritonitis, as the effluent was cloudy. Peritoneal fluid cell count revealed 3520 nucleated cells, 41 red blood cells, 91% neutrophils, 1% lymphocyte, 8% eosinophils, and gram stain revealed more than 100 white blood cells/low-power field; no organisms were seen. The patient was started on empiric intraperitoneal amikacin and ceftriaxone. The effluent culture from aerobic bottle grew *N farcinica*, identified by MALDI-TOF (matrix-assisted desorption ionization time-of-flight) technique. Oral linezolid was added to the antibiotic regimen after the organism was identified. The susceptibilities are mentioned in Table 1. The general surgery team removed the peritoneal dialysis catheter. Computed tomography of the head, chest, abdomen, and pelvis was done for suspected disseminated nocardiosis, which revealed discrete noncalcified mass measuring 3.3 cm in the left apex. Mixed density masses identified in the right lower abdominal quadrants of 4.1 cm and 5.9 cm that could represent infectious or prior resolving retroperitoneal hematoma. The patient’s family opted against any aggressive interventions, including the biopsy of the lung mass, and it was decided to treat with oral linezolid for 6 months. The patient passed away, unfortunately, despite treatment as the clinical condition deteriorated.

**Table 1.** Susceptibilities of *Nocardia farcinica*.

Antibiotic	Minimal Inhibitory Concentration, µg/mL	Sensitive (S), Resistant (R), or Intermediate (I)
Amikacin	≤0.5	S
Amoxicillin + clavulanate	4	S
Cefepime	>32	R
Cefotaxime	32	I
Ceftriaxone	32	I
Ciprofloxacin	>2	R
Clarithromycin	>16	R
Doxycycline	8	R
Imipenem	2	S
Linezolid	2	S
Minocycline	4	I
Moxifloxacin	2	I
Tobramycin	16	R
Trimethoprim-sulfamethoxazole	≤1/20	S

## Discussion

*Nocardia* species are aerobic, weakly gram-positive, partially acid-fast, methenamine silver-positive aerobic actinomycete. The bacterium is characterized by filamentous branches measuring less than 1 µm thick and are not identified by conventional staining.^4^

*Nocardia* contains more than 100 species and is widely distributed in the environment in water, soil, decaying vegetation, and organic matter.^5^ The most important species implicated in human diseases is *Nocardia* *asteroids*. Other clinically relevant species are *N brazeliensis, N farcinica, N nova, N pseudobrasiliensis*, and *N transvalensis*.^4^ Accurate identification of species is essential because of the difference in the spectrum of diseases that they can cause and variations in their antimicrobial susceptibilities.

*Nocardia farcinica* is an uncommon cause for nocardiosis, particularly in immunocompetent individuals. It frequently involves adult males, but cases have been reported in children and females. It can cause nonspecific symptoms and signs, which often makes the early diagnosis difficult. Most common routes of infection are by inhalation or by percutaneous inoculation.^5^ Patients who have underlying malignancy, human immunodeficiency virus, dialysis dependence, receiving chemotherapy, and autoimmune disease are susceptible to *N farcinica* infections. It can cause multi-organ involvement and can cause life-threatening infections.^5^

*Nocardia farcinica* was infrequently isolated but could be related to the underdiagnosis of the organism. Recent changes in diagnostic technologies made the organism to be isolated more. It is highly virulent and is known to be naturally resistant to multiple antibiotics.^4^ Prompt diagnosis and timely treatment can be lifesaving.

*Nocardia farcinica* is a very slow-growing organism and can take up to more than 5 days to be identified. The blood cultures in patients with *N farcinica* infection are frequently negative as in our patient. Early diagnosis is made by the tissue culture, phenotypic and molecular methods, and 16S rRNA (recombinant ribonucleic acid) gene sequencing technique.^1^

The susceptibilities for antibiotics are specific for *N farcinica*, but is often resistant to commonly used antibiotics as in our patient. Most commonly used antibiotic is trimethoprim-sulfamethoxazole, but usually a combination of antibiotics like amikacin, moxifloxacin, imipenem-cilastatin, trimethoprim-sulfamethoxazole, and linezolid is needed.^1,4,5^ In patients with disseminated infections, prolonged treatment is required ranging from 6 months to 1 year.^1^ Despite aggressive therapy, the mortality has been reported as high as 39%.^4^

## Conclusion

This case highlights that rare organisms like *N farcinica* can cause peritonitis. Recently, advances in microbiological identification techniques has aided in isolating the organism. This case report also discusses the virulence and mortality of the organism.
